# P-8. Determining the Optimal Duration of Therapy for Bloodstream Infections Caused by Obstructing Ureteral Stones

**DOI:** 10.1093/ofid/ofaf695.239

**Published:** 2026-01-11

**Authors:** Chloe Kotrba, Allison Gibble, Kelsey Zeeck, Sara Revolinski

**Affiliations:** Froedtert and the Medical College of Wisconsin, Milwaukee, WI; Froedtert & the Medical College of Wisconsin, Milwaukee, WI; Froedtert and the Medical College of Wisconsin, Milwaukee, WI; Froedtert and the Medical College of Wisconsin, Milwaukee, WI

## Abstract

**Background:**

Currently, variability exists in determining the duration of antibiotic therapy when treating patients for bacteremia secondary to obstructive urinary stones. Patients are treated with either a prolonged course of antibiotics through stone removal or a defined course of antibiotics for bacteremia. The purpose of this study was to determine the optimal duration of antibiotics for bacteremia associated with infected urinary stones.

**Methods:**

This retrospective cohort study evaluated adult patients admitted with bacteremia due to obstructive urinary stones. Eligible patients underwent both urgent urinary stenting at the time of bacteremia identification and subsequent stone removal weeks later at a Froedtert site. Patients were placed into two groups during data review. The antibiotic free group was treated initially for bacteremia followed by a time off antibiotics prior to stone removal. The continued group was on antibiotics through stone removal. The primary composite outcome was incidence of treatment failure (recurrent bacteremia or antibiotic re-initiation) from stenting to stone removal. Additional outcomes included days treated with antibiotics, time from stenting to stone removal, and infection related readmissions.

**Results:**

41 patients were included in the antibiotic free group and 22 patients in the continued. The primary outcome was met in 6 of 41 (14.6%) patients in the antibiotic free group, compared to the continued group, 2 of 22 (9.1%) (p=0.702). The average number of days treated with antibiotics was 13.8 (± 3.1) in the antibiotic free group, compared to the continued group, 20.1 (± 6.0) (p value = < .00001). The antibiotic free group had an average of 50 days (± 33.5) from stenting to stone removal, compared to the continued group, 18 days (± 6.5) (p value = .00003). A subgroup of the antibiotic free group with stone removal < 6 weeks from stenting was examined. This subgroup met the primary outcome in 2 of 23 (8.7%) patients. Infection related re-admissions were similar in this subgroup (2/23, 8.7%) and the continued group (2/22, 9.1%).

**Conclusion:**

In patients with bacteremia secondary to obstructing urinary stones, treatment outcomes were similar in prolonged antibiotic courses compared to defined courses of antibiotics, if stone removal was < 6 weeks from stenting.

**Disclosures:**

All Authors: No reported disclosures

